# Neurochemical and motor changes in mice with combined mutations linked to Parkinson’s disease

**DOI:** 10.1080/20010001.2017.1267855

**Published:** 2017-01-05

**Authors:** Xiang Bai, Margaret Chia-Ying Wey, Paul Anthony Martinez, Chao Shi, Elizabeth Fernandez, Randy Strong

**Affiliations:** ^a^ Department of Pharmacology, University of Texas Health Science Center at San Antonio, San Antonio, TX, USA; ^b^ Barshop Institute for Longevity and Aging Studies, University of Texas Health Science Center at San Antonio, San Antonio, TX, USA; ^c^ Department of Management Science and Statistics, College of Business, University of Texas at San Antonio, San Antonio, TX, USA; ^d^ Geriatric Research, Education and Clinical Center, South Texas Veterans Health Care Network, San Antonio, TX, USA

**Keywords:** Glutathione peroxidase-1 (Gpx1), aldehyde dehydrogenase-1a1 (Aldh1a1), Parkinson’s disease, knockout mice, lipid peroxidation, oxidative stress, motor deficit, dopamine and its metabolites, movement disorder, aldehyde

## Abstract

Considerable evidence suggests that oxidative stress plays a role in the pathogenesis of Parkinson’s disease (PD), the most prevalent neurodegenerative movement disorder. Reduced expression of aldehyde dehydrogenase-1 (ALDH1) and glutathione peroxidase-1 (GPX1), enzymes that function to detoxify aldehydes and hydroxyl radicals, respectively, has been reported in the substantia nigra of patients who died with PD. To determine whether deficiency in these two genes contributes to the pathogenesis of PD, mice were generated with homozygous null mutations of both Aldh1a1 (the murine homolog of ALDH1) and Gpx1 genes [knockout (KO) mice]. At 6 and 18 months of age, KO mice showed a significantly decreased latency to fall in the automated accelerating rotarod test and increased time to complete the pole test opamine levels were not altered; however, the dopamine metabolite 3,4-dihydroxyphenylacetic acid (DOPAC) and the DOPAC/dopamine ratio were significantly reduced at 18 months of age. Proteins adducted with 4-hydroxynonenal, the end-product of lipid peroxidation, were increased in the. midbrain and striatum of KO mice at 6 and 18 months. In conclusion, dual mutations in Gpx1 and Aldh1a1 genes are associated with motor deficits and increased lipid peroxidation in adult mice.

## Introduction

Parkinson’s disease (PD) is the second most prevalent age-related neurodegenerative disorder, after Alzheimer’s disease, affecting up to 5% of the population aged 65–85 years. PD is characterized by progressive degeneration of dopaminergic neurons in the substantia nigra (SN) and dopamine depletion in the nigrostriatal pathway. Symptoms of PD include postural instability, rigidity, bradykinesia, and tremor. The pathogenesis of PD is still largely unclear.

A large amount of evidence suggests that oxidative stress plays an important role in the pathogenesis of neurodegenerative diseases including PD [1]. Free radicals and biogenic aldehydes are major sources of oxidative damage. Glutathione peroxidase (GPX) is a key enzyme in removing free radicals *in vivo*. It protects cells against oxidative damage by oxidizing glutathione to glutathione disulfide. Decreased levels of the antioxidant glutathione in the SN have been reported in patients who died with PD [2]. GPX1, the most abundant form of GPX, plays a crucial role in protecting cells against oxidative damage by decreasing the production of reactive hydroxyl radicals [3]. GPX1 messenger ribonucleic acid (mRNA) expression is significantly reduced in the SN of PD patients [4]. Intrastriatal injection of a Gpx1-overexpressing lentivirus is neuroprotective in mice treated with 6-hydroxydopamine [5]. Aldehyde dehydrogenases (ALDHs) are a family of enzymes that detoxify biogenic aldehydes *in vivo*. Aldehydes are particularly destructive because they have relatively long half-lives, allowing the aldehydes to accumulate at the site of injury to injure healthy cells. They may also cross cell membranes, leading to damage distal to the site of injury. Elevated levels of 3,4-dyhydroxyphenylacetaldehyde (DOPAL), a neurotoxic metabolite of dopamine, are implicated in PD [6,7]. Moreover, 4-hydroxynonenal (4-HNE) is elevated in the brains of patients who had PD [8]. Both DOPAL and 4-HNE are toxic to dopaminergic cells *in vitro* and *in vivo* [6,9–12]. The cytosolic aldehyde dehydrogenase ALDH1 is specifically and richly expressed in dopaminergic neurons in the SN [13–15] and its mRNA expression is significantly decreased in surviving dopaminergic neurons in the SN of PD patients [14]. The above evidence suggests that increased oxidative stress in PD may result at least partially from combined deficits in ALDH1 and GPX1.

Mice lacking only Gpx1 have normal locomotor activity at 6 and 18 months [16], normal performance on the rotarod at 6 and 18 months [16], and normal performance on the pole test at 13 months of age (unpublished data). To further investigate the contribution of dual deficiencies in GPX1 and ALDH1 to the pathogenesis of PD, we cross-bred mice with null mutations in Gpx1 and Aldh1a1 genes and characterized their motor performance and neurochemistry as a function of age. We found that mice deficient in both Gpx1 and Aldh1a1 exhibited increased 4-HNE, reduced 3,4-dihydroxyphenylacetic acid (DOPAC)/dopamine ratios, and motor deficits.

## Materials and methods

### Animals

Mice with homozygous deletions of both Aldh1a1 and Gpx1 genes [knockout (KO) mice] on a C57BL/6 background were generated and genotyped as described previously [17]. In brief, the KO mice were generated by crossing mice with a targeted mutation in Aldh1a1 with a line of mice carrying a mutation in Gpx1. The Aldh1a1 mutant mice were generated by Gregg Duester and colleagues using a targeted deletion at exon 11 of the Aldh1a1 allele, and were backcrossed by us for eight generations to C57BL/6J. Aldh1a1^–/–^ mice were crossed with Gpx1^–/–^ mice, which had been backcrossed to 10 generations to C57Bl/6 mice, to produce mice heterozygous for both genes, Aldh1a1^+/–^ × Gpx1^+/–^. Cross-breeding of Aldh1a1^+/–^ × Gpx1^+/–^ generated the Aldh1a1^–/–^ × Gpx1^–/–^ line (i.e. homozygous null for both genes, KO) and the wild-type Aldh1a1^+/+^ × Gpx1^+/+^ line (WT). Thus, the genetic backgrounds of the WT line and the homozygous null line were identical. The two lines were maintained by breeding male and female Aldh1a1^–/–^ × Gpx1^–/–^ or Aldh1a1^+/+^ × Gpx1^+/+^ mice. Age-matched male KO and corresponding WT littermate control mice were used for the behavioral tests and neurochemical assays. Animal experiments were conducted according to the National Institutes of Health ‘Guide for the Care and Use of Laboratory Animals’ and were approved by the Institutional Animal Care and Use Committee of the University of Texas Health Science Center at San Antonio.

### Behavioral tests

The following behavioral tests were chosen because they are widely used to evaluate motor function in mouse models of PD. The pole test and the accelerating rotarod are both sensitive to deficits in nigrostriatal dopamine. The pole test was devised as a measure of bradykinesia, one of the diagnostic criteria for PD [18]. It is sensitive to reductions in dopamine in the nigrostriatal pathway in the 1-methyl-4-phenyl-1,2,5,6-tetrahydropyridine (MPTP)-induced mouse model of PD. The accelerating rotarod test measures changes in balance and coordination, locomotor functions that are impaired in PD [19,20]. It is sensitive to reductions in nigrostriatal dopamine in MPTP-treated mice [19].

#### Accelerating rotarod test

Mice were pretrained for 5 days on the rotarod apparatus Rotamex 4/8 (Columbus Instruments, Columbus, OH). Three trials were performed on each day. On each trial, the rotarod started at an initial speed of 4 rpm and then increased to 40 rpm within 300 s. The average latency to fall from the accelerating rod was calculated from three trials on the test day.

#### Pole test

Each mouse was placed head-up on top of a vertical wooden pole 50 cm long (1 cm in diameter) which was placed in the home cage. When placed on the pole, animals orient downward and descend the length of the pole back into their home cage. Mice received 2 days of training consisting of three trials per day. On the test day, mice received three trials, and the time to reverse was recorded.

#### Grip strength test

Each mouse was allowed to hold on to the metal grid of a grip meter (Columbus Instruments, Columbus, OH) with their forelimbs. The experimenter then gently pulled the mouse backwards by the tail until it lost its grip. Five trials were performed and the peak grip strength was recorded as pounds of force (LBF).

### Preparation of brain tissue

Brains were rapidly collected following brief carbon dioxide anesthesia and decapitation. For neurochemical assays, brain tissue was rapidly dissected on an ice-cold glass plate; tissue was then snap-frozen on dry ice and transferred to a –80°C freezer for storage until assayed.

### High-performance liquid chromatography

Levels of dopamine and its metabolites, 3,4-dihydroxyphenylacetic acid (DOPAC) and homovanillic acid (HVA), were measured by high-performance liquid chromatography (HPLC) electrochemical detection as described previously [7]. Samples were homogenized in ice-cold 0.1 M HClO_4_ containing 10 ng/ml 3,4-dihydroxybenzylamine (DHBA) as the internal standard and centrifuged to remove precipitated proteins. The supernatants were used for HPLC analysis. An aliquot of the supernatant was filtered through a 0.45 mm microcentrifuge filter (Millipore, Temecula, CA, USA). Dopamine, serotonin, and metabolites were separated using an HPLC (ESA, North Billerica, MA, USA) system with an HR-80 reverse-phase C18 column (4.6680 mm). Samples were detected with an electrochemical detector on a dual-electrode analytical cell (Model 5011A; Thermo Scientific, Franklin, MA, USA). The pH of the 6% acetonitrile (v/v) mobile phase was adjusted to pH 3.11 with phosphoric acid after the addition of organic modifiers. The flow rate of the mobile phase was set at 1.0 ml/min.

### Western blot analysis of 4-HNE-adducted proteins

Protein from frozen striatum and midbrain was extracted with 1 × radioimmunoprecipitation assay buffer (CST, Beverly, MA, USA) with 1 × Calbiochem Protease Inhibitor Cocktail Set I and 1 × Halt* Phosphatase Inhibitor Cocktail (Thermo Scientific). A Tissue Lyser LT (Invitrogen, Pleasanton, CA, USA) with a 5 mm steel bead was used to homogenize tissues at 50 Hz. Tissue lysate was then centrifuged at 13,000 rpm for 15 min at 4°C. Supernatant was saved in a –80°C freezer. Bradford protein assay reagents (Bio-Rad, Irvine, CA, USA) were used to determine protein concentration. Protein (40 μg/lane) was then separated on a 4–12% Criterion sodium dodecyl sulfate–polyacrylamide gel electrophoresis gel (Bio-Rad) and transferred on to nitrocellulose membrane. Proteins were detected with antibodies directed specifically to 4-HNE protein adducts (R&D, Minneapolis, MN, USA) and glyceraldehyde-3-phosphate dehydrogenase (GAPDH) (CST), respectively, in the midbrain and striatum of 6- and 18-month-old mice. An appropriate secondary antibody from LiCor (IR800 or IR680) was used corresponding to each primary antibody. Immunoreactive bands were imaged and quantified using Odyssey software (LiCor, Lincoln, NE, USA).

### Statistical analyses

Results for each group were summarized and expressed as the mean ± SEM. Data were analyzed using a general linear model with the statistical software SAS 9.0. The Tukey–Kramer method was used for adjustment for multiple comparisons. Differences were considered statistically significant when *p* < 0.05.

## Results

### Effect of genotype and age on rotarod performance

**Figure 1.**
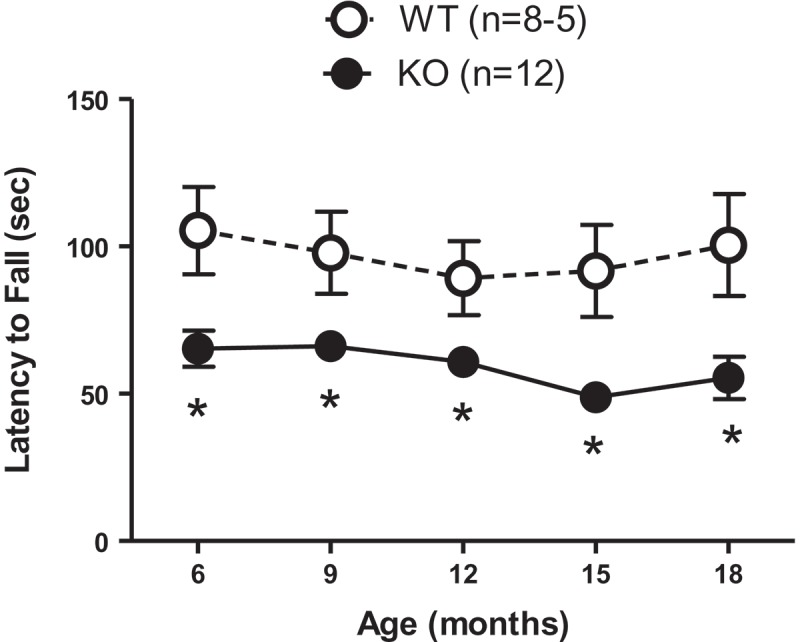
Aldh1a1/Gpx1 knockout (KO) mice show significantly reduced mean latency to fall from the rotarod compared to age-matched wild-type (WT) mice. Data are expressed as the mean ± SEM of the number of mice in parentheses. Data were analyzed using a general linear model. The Tukey–Kramer method was used for adjustment for multiple comparisons. *p < 0.05, WT vs KO at the indicated time-points.

Mice were tested for rotarod performance at 3 month intervals, beginning at 6 months of age. Figure 1 shows that there was an effect of genotype (*p* **< 0.0001), but no effect of age (*p* **> 0.05) in either the WT or KO group. The Aldh1a1/Gpx1 KO group exhibited a significantly reduced mean latency to fall from the rotarod compared to the age-matched WT control group at each age (6–18 months). The least squares mean latency to fall for the KO group was 59.30 s, with a 95% confidence interval (CI) of 52.20 to 66.40. The least squares mean latency to fall for the WT group was 96.92 s (95% CI 87.45, 106.39).

### Effect of genotype and age on performance on the pole test

**Figure 2.**
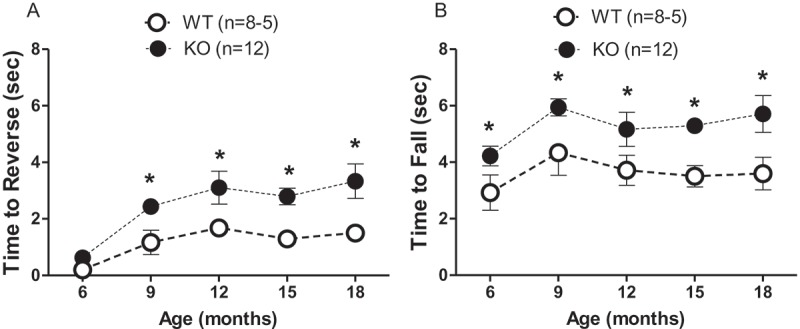
Aldh1a1/Gpx1 knockout (KO) mice show significantly increased time to fall and time to reverse in the pole test compared to age-matched wild-type (WT) mice. (A) Time to reverse; (B) time to fall. Data are expressed as the mean ± SEM of the number of mice in parentheses. Data were analyzed using a general linear model. The Tukey–Kramer method was used for adjustment of multiple comparisons. *p < 0.05, WT vs KO at the indicated time-points.

Consistent with performance in the accelerating rotarod test, deletion of both Aldh1a1 and Gpx1 genes resulted in motor deficits in the pole test, as evidenced by the increased time to reverse (Figure 2(a)) and time to fall (Figure 2(b)) of KO mice in the pole test compared to age-matched WT control mice tested at the same five time-points from 6 to 18 months of age (genotype effect: *p *< 0.0001). The least squares means of the time to reverse were 2.46 s (95% CI 2.14, 2.78) for KO and 1.17 s (95% CI 0.74, 1.60) for WT. The least squares means of the time to fall were 5.27 s (95% CI 4.85, 5.68) for KO and 3.61 s (95% CI 3.05, 4.18) for WT. Age had no significant effect on the time to reverse or the time to fall in KO mice (age effect: *p* **> 0.05).

### Effect of genotype and age on grip strength and body weight

**Figure 3.**
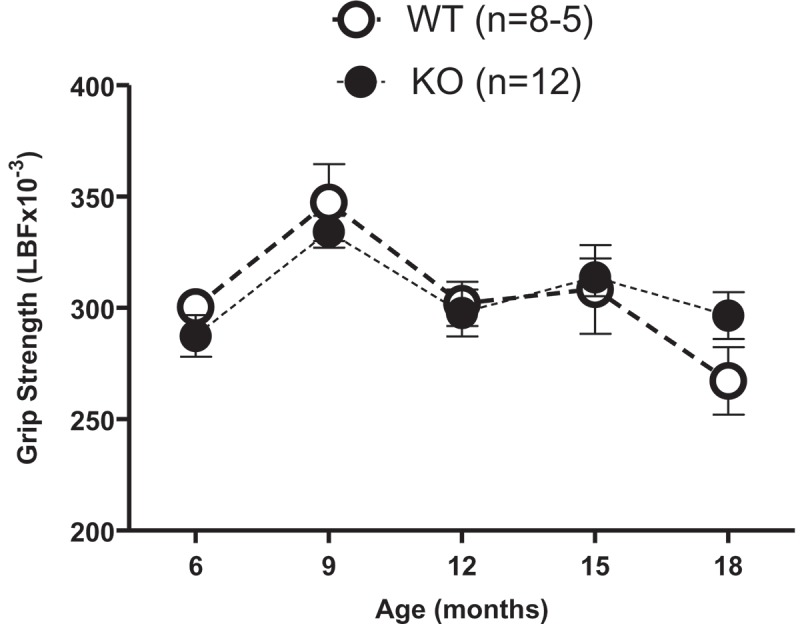
Aldh1a1/Gpx1 knockout (KO) mice do not show altered grip strength compared to age-matched wild-type (WT) mice. Data are represented as the mean ± SEM of the number of animals in parentheses. Data were analyzed using a general linear model. The Tukey–Kramer method was used for adjustment of multiple comparisons. *p* < 0.05, significant effect of age. No significant effect of genotype. LBF = pounds of force.

**Figure 4.**
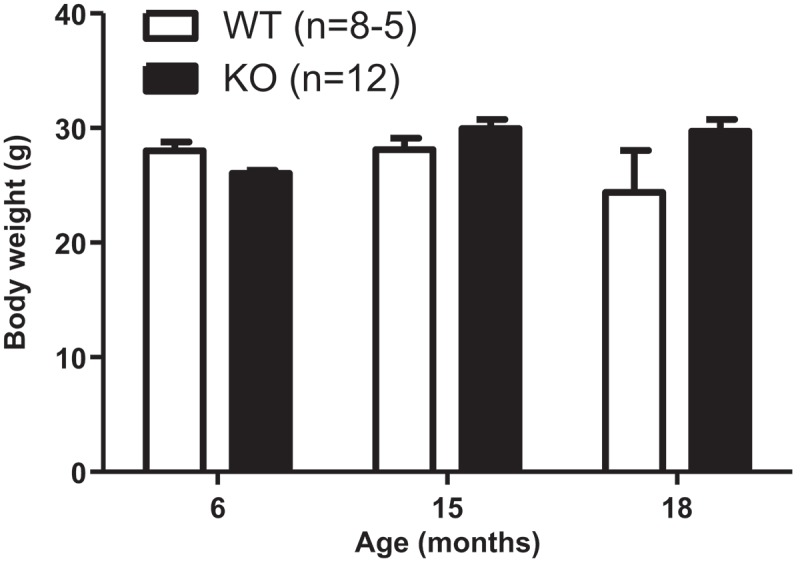
Aldh1a1/Gpx1 knockout (KO) mice do not show altered body weight compared to age-matched wild-type (WT) mice. Data in each group are represented as the mean ± SEM. Data were analyzed using a general linear model. The Tukey–Kramer method was used for adjustment of multiple comparisons.

Deletion of both Aldh1a1 and Gpx1 genes did not alter grip strength compared to age-matched WT control mice at 6 or 18 months of age (Figure 3) (genotype effect: *p* **> 0.05), although aging from 6 to 18 months decreased grip strength in both WT and KO mice (age effect: *p* **< 0.05). The least squares means of grip strength were 305.95 LBF × 10^–^^3^ (95% CI 297.19, 314.72) for KO and 305.03 LBF × 10^–^^3^ (95% CI 293.30, 316.75) for WT. We also found that KO mice had similar body weights to age-matched WT control mice (Figure 4) (genotype effect: *p* **> 0.05). The least squares means of body weight were 28.58 g (95% CI 27.33, 29.83) for KO and 26.84 g (95% CI 25.14, 28.53) for WT. Age did not change body weight in the age range tested (age effect: *p* **> 0.05).

### Effect of genotype on dopamine and its metabolites

**Figure 5.**
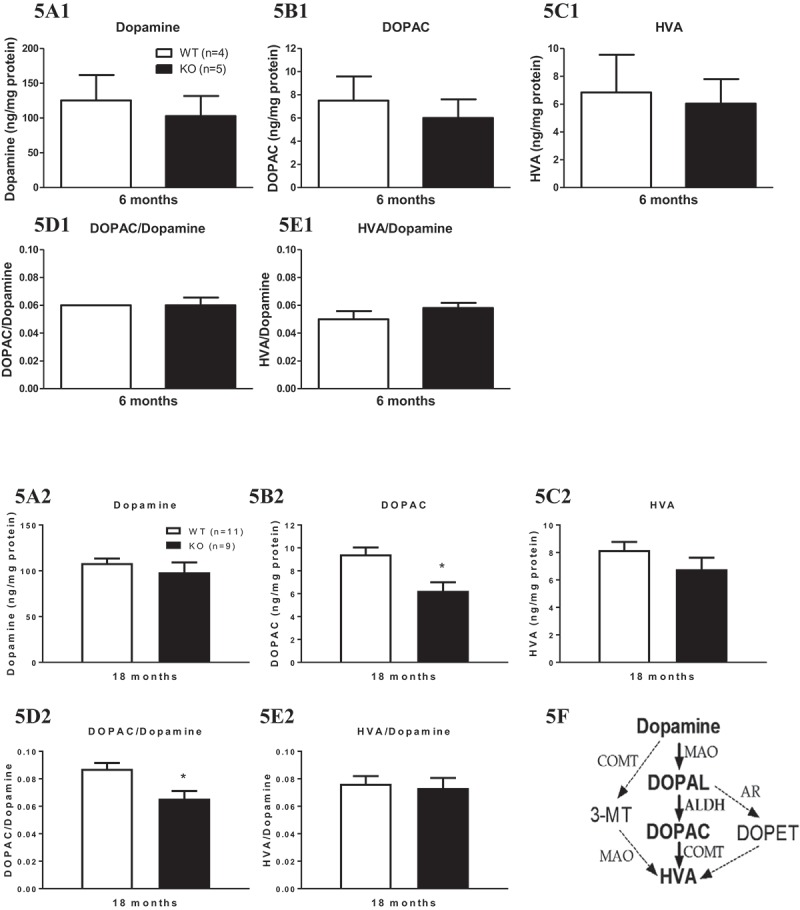
Aldh1a1/Gpx1 knockout (KO) mice show significantly reduced 3,4-dihydroxyphenylacetic acid (DOPAC) and the DOPAC/dopamine ratio in striatum at 18 months of age. Data in each group are expressed as the mean ± SEM of the number of animals in parentheses. Data were analyzed using a general linear model. The Tukey–Kramer method was used to adjust for multiple comparisons. *p < 0.05, WT vs KO. HVA = homovanillic acid; DOPAL = 3,4-dyhydroxyphenylacetaldehyde; COMT = catechol-*O*-methyltransferase; 3-MT = 3-methoxytyramine; MAO = monoamine oxidase; ALDH = aldehyde dehydrogenase.

Levels of striatal dopamine (Figure 5(A1,A2)), its metabolite HVA (Figure 5(C1,C2)), and the HVA/dopamine ratio (Figure 5(E1,E2)) were not altered by deletion of Aldh1a1 and Gpx1 genes at either age. However, levels of the dopamine metabolite DOPAC (Figure 5(B1,B2)) and the DOPAC/dopamine ratio (Figure 5(D2)) were significantly reduced by deletion of both of the genes (genotype effect: *p* **< 0.05) at 18 months of age. Levels of striatal DOPAC in 18-month-old KO mice were decreased by 30.88% compared to the WT group (95% CI 25.87, 37.98). The DOPAC/dopamine ratio in the striatum of 18-month-old KO mice was decreased by 19.29% compared to the WT group (95% CI 17.14, 21.94).

### Effect of genotype on 4-HNE-adducted proteins

**Figure 6.**
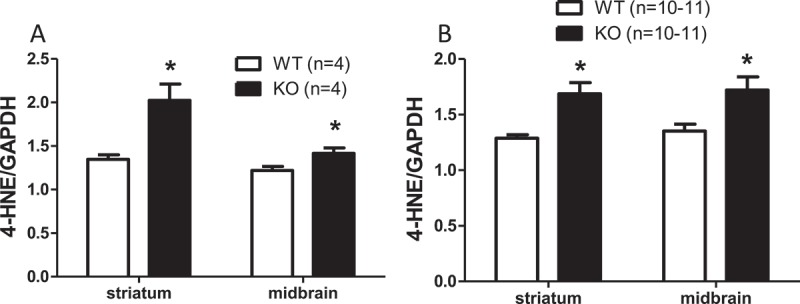
Aldh1a1/Gpx1 knockout (KO) mice show significantly increased 4-hydroxynonenal (4-HNE) in striatum and midbrain compared to age-matched wild-type (WT) mice. (A) 6-month-old mice; (B) 18-month-old mice. Data in each group are expressed as the mean ± SEM of the number of animals in parentheses. Data were analyzed using a general linear model. The Tukey–Kramer method was used to adjust for multiple comparisons. *p < 0.05, WT vs KO at the indicated time-point. GAPDH = glyceraldehyde-3-phosphate dehydrogenase.

Protein adducted to 4-HNE was significantly elevated in the midbrain and striatum of 6-month-old (Figure 6(a) and Figure S1) and 18-month-old (Figure 6(b) and Figure S2) KO mice compared to age-matched WT control mice. There was a significant effect of genotype: (*p* **< 0.05). Levels of 4-HNE protein adducts in KO mice were increased by 21.92% (95% CI 19.49, 25.04) in the midbrain and by 40.93% (95% CI 36.7, 46.24%) in the striatum.

## Discussion

Expression of ALDH1 and GPX1, gene products that are important for the clearance of aldehydes and free radicals, respectively, was reported to be reduced in the SN of PD patients, suggesting that ALDH1 and GPX1 play important roles in the pathogenesis of PD [4,14]. In the current study, we found that homozygous null mutations of both Aldh1a1 and Gpx1 genes in mice resulted in motor deficits and oxidative stress, as evidenced by significantly decreased latency to fall in the automated accelerating rotarod test, time to reverse and time to fall in the pole test, and significantly increased levels of the lipid peroxidation end-product 4-HNE in the midbrain and striatum. Deficits in motor function of KO mice on the rotarod and pole tests compared to WT mice were not due to reduced grip strength or increased body weight because there were no differences in grip strength or body weight between the WT and KO mice. The lack of age-related deficits in motor function in WT mice is consistent with the results of our previous study in mice with a similar C57BL/6 genetic background [7].

It is not clear whether Gpx1 or Aldh1a1 plays the more important role in the PD-like motor phenotypes observed. Homozygous deletion of both genes causes notable motor deficits on the accelerating rotarod but deletion of either gene by itself does not [16; unpublished data]. Moreover, mice with homozygous deletion of just the Gpx1 gene have normal locomotor activity in a novel environment and normal performance on the rotarod at the ages of 6 and 18 months [16]. In addition, we found that 13-month-old Gpx1 knockout mice perform normally on the pole test, as evidenced by a similar time to reverse and a similar time to fall, and on the rotarod test, as evidenced by an unchanged latency to fall compared to WT mice (unpublished data). This evidence suggests that deletion of both Aldh1a1 and Gpx1 genes may have additive effects on motor dysfunction.

Elevated 4-HNE protein adducts in the midbrain and striatum in the present study may result from deficiency in both Aldh1a1 and Gpx1 genes. Based on our data, we cannot infer whether other forms of oxidative damage are altered by deletion of Aldh1a1 and Gpx1 genes, including protein carbonyls, F2-isoprostanes, and 8-*oxo*-deoxyguanosine (DNA oxidative damage). So, it is still not clear whether deficiency in both Aldh1a1 and Gpx1 genes leads specifically to lipid peroxidation or more generally to oxidative damage, and it is not clear whether increased 4-HNE in brain can, by itself, lead to motor deficits in mice.

We found significantly reduced striatal levels of the dopamine metabolite DOPAC in Aldh1a1^(–/–)^ × Gpx1^(–/–)^ mice at 18 months of age. This may be due to deficiency in Aldh1a1 alone or to the combined reduction in Aldh1a1 and Gpx1 expression. Dopamine is metabolized to DOPAL by monoamine oxidase (MAO), and then metabolized to DOPAC by ALDH (Figure 5(f)). Therefore, deficiency of Aldh1a1 alone may be expected to cause decreased DOPAC content. However, others have reported no significant changes in dopamine turnover, as measured by the reduced DOPAC/dopamine ratio in KO mice lacking Aldh1a1 [21]. The other ALDH present in the nigrostriatal pathway is Aldh2. It has been reported that products of oxidative stress, such as 4-HNE, at physiological levels, inhibit ALDH activity, as measured by the biotransformation of DOPAL to DOPAC [22]. Thus, it is possible that increased oxidative stress resulting from reduced Gpx1 expression may inhibit Aldh2 activity and, together with reduced expression of Aldh1a1, this may result in decreased conversion of DOPAL to DOPAC. Since DOPAL is known to be neurotoxic, it can contribute to synaptic degeneration. The findings of the current study are consistent with the results of our previous study of mice with mutations in both Aldh1a1 and Aldh2 [7]. In that study, we measured age-related decreases in DOPAC content and DOPAC/dopamine ratios and increases in DOPAL. In contrast to the findings in the present study, we saw significant decreases in striatal dopamine in that study, beginning in midlife. One possible explanation for these findings is that the loss of DOPAC and reduction in the DOPAC/dopamine ratio are relatively early events in PD and that the loss of dopamine occurs later in life in this animal model, owing to the well-known ability of dopamine neurons to compensate for damage to dopamine terminals by increasing the synthesis of dopamine in surviving terminals [23,24]. Indeed, in our previous study, decreases in DOPAC and increases in DOPAL preceded the age-related loss of dopamine and loss of dopaminergic neurons [7]. The loss of DOPAC and the deficits we observed in performance on dopamine-sensitive motor tests in the present study are consistent with such a possibility. We plan to investigate this possibility further in future studies.

The results from our study support a role for reduced expression of both ALDH1 and GPX1 in the pathogenesis of PD. These findings also suggest that ALDH1 and GPX1 may be potentially important therapeutic targets for PD. It was reported that reactive aldehydes can react with and be chemically inactivated by hydroxylamines [11]. Also, *N*-acetylcysteine has been shown to be neuroprotective by increasing glutathione and enhancing antioxidant defense by elevating the activity of antioxidant enzymes such as GPX1 [25]. Taken together with the results from the current study, these findings indicate that combined use of aldehyde-trapping agents and cysteine-containing compounds may provide a new therapeutic approach for intervention in PD.

Pesticide exposure and gene–environment interactions play crucial roles in the pathogenesis of PD [26,27]. Gpx1 null mice are more susceptible to oxidative stress-inducing agents such as paraquat and hydrogen peroxide [28]. Thus, it would be interesting to know whether the mouse with homozygous deletion of Gpx1 and Aldh1a1 genes is more susceptible to neurotoxins such as paraquat, rotenone, or MPTP. We previously reported that rapamycin improves motor performance in the A53T human alpha-synuclein mouse model of PD. Rapamycin treatment was associated with a reduction in 4-HNE-adducted protein and with decreased synaptic injury in the nigrostriatal pathway [29]. Our results suggest that further investigation is warranted into whether combining aldehyde-trapping agents such as hydralazine with *N*-acetylcysteine or rapamycin would be neuroprotective and improve motor function in PD.

In summary, we have shown that mice carrying homozygous null mutations in both Gpx1 and Aldh1a1 genes exhibit PD-like motor deficits, a reduced DOPAC/dopamine ratio, and increased 4-HNE-adducted proteins in adult mice. Our study is innovative in that we have used mice with deletion of both Gpx1 and Aldh1a1 genes to study the contribution of two sources of oxidative stress to characteristic manifestations of PD. This is the first study to examine whether the deficits previously reported in both Gpx1 and Aldh1a1 expression in the brains of patients who died with PD may be related to the pathogenesis of this disease. Our results indicate that mice homozygous null for Gpx1 and Aldh1a1 may provide a useful model to study the pathogenesis of PD and identify new therapeutic strategies.

## Supplementary Material

PBA_33852_Bai_Supplementary_figure.docx

## References

[CIT0001] Zarkovic K. (2003). 4-Hydroxynonenal and neurodegenerative diseases. Mol Aspects Med.

[CIT0002] Fitzmaurice PS, Ang L, Guttman M (2003). Nigral glutathione deficiency is not specific for idiopathic Parkinson’s disease. Mov Disord.

[CIT0003] Halliwell B (1992). Reactive oxygen species and the central nervous system. J Neurochem.

[CIT0004] Duke DC, Moran LB, Pearce RK (2007). The medial and lateral substantia nigra in Parkinson’s disease: mRNA profiles associated with higher brain tissue vulnerability. Neurogenetics.

[CIT0005] Ridet JL, Bensadoun JC, Deglon N (2006). Lentivirus-mediated expression of glutathione peroxidase: neuroprotection in murine models of Parkinson’s disease. Neurobiol Dis.

[CIT0006] Mattammal MB, Haring JH, Chung HD (1995). An endogenous dopaminergic neurotoxin: implication for Parkinson’s disease. Neurodegeneration.

[CIT0007] Wey MC, Fernandez E, Martinez PA (2012). Neurodegeneration and motor dysfunction in mice lacking cytosolic and mitochondrial aldehyde dehydrogenases: implications for Parkinson’s disease. Plos One.

[CIT0008] Castellani RJ, Perry G, Siedlak SL (2002). Hydroxynonenal adducts indicate a role for lipid peroxidation in neocortical and brainstem Lewy bodies in humans. Neurosci Lett.

[CIT0009] Burke WJ, Li SW, Chung HD (2004). Neurotoxicity of MAO metabolites of catecholamine neurotransmitters: role in neurodegenerative diseases. Neurotoxicology.

[CIT0010] Lamensdorf I, Eisenhofer G, Harvey-White J (2000). Metabolic stress in PC12 cells induces the formation of the endogenous dopaminergic neurotoxin, 3,4-dihydroxyphenylacetaldehyde. J Neurosci Res.

[CIT0011] Wood PL, Khan MA, Kulow SR (2006). Neurotoxicity of reactive aldehydes: the concept of ‘aldehyde load’ as demonstrated by neuroprotection with hydroxylamines. Brain Res.

[CIT0012] Panneton WM, Kumar VB, Gan Q (2010). The neurotoxicity of DOPAL: behavioral and stereological evidence for its role in Parkinson disease pathogenesis. Plos One.

[CIT0013] McCaffery P, Drager UC (1994). High levels of a retinoic acid-generating dehydrogenase in the meso-telencephalic dopamine system. Proc Natl Acad Sci USA.

[CIT0014] Galter D, Buervenich S, Carmine A (2003). ALDH1 mRNA: presence in human dopamine neurons and decreases in substantia nigra in Parkinson’s disease and in the ventral tegmental area in schizophrenia. Neurobiol Dis.

[CIT0015] Westerlund M, Galter D, Carmine A (2005). Tissue- and species-specific expression patterns of class I, III, and IV Adh and Aldh 1 mRNAs in rodent embryos. Cell Tissue Res.

[CIT0016] Hennis MR, Marvin MA, Taylor CM (2014). Surprising behavioral and neurochemical enhancements in mice with combined mutations linked to Parkinson’s disease. Neurobiol Dis.

[CIT0017] Bai X, Fermandez E, Gould G (2013). Homozygous deletion of glutathione peroxidase 1 and aldehyde dehydrogenase 1a1 genes is not associated with schizophrenia-like behavior in mice. J Biochem Pharmacol Res.

[CIT0018] Ogawa N, Hirose Y, Ohara S (1985). A simple quantitative bradykinesia test in MPTP-treated mice. Res Commun Chem Pathol Pharmacol.

[CIT0019] Rozas G, López-Martín E, Guerra MJ (1998). The overall rod performance test in the MPTP-treated-mouse model of Parkinsonism. J Neurosci Methods.

[CIT0020] Boonstra TA1, van der Kooij H, Munneke M (2008). Gait disorders and balance disturbances in Parkinson’s disease: clinical update and pathophysiology. Curr Opin Neurol.

[CIT0021] Anderson DW, Schray RC, Duester G (2011). Functional significance of aldehyde dehydrogenase ALDH1A1 to the nigrostriatal dopamine system. Brain Res.

[CIT0022] Doorn JA, Florang VR, Schamp JH (2014). Aldehyde dehydrogenase inhibition generates a reactive dopamine metabolite autotoxic to dopamine neurons. Parkinsonism Relat Disord.

[CIT0023] Mogi M, Harada M, Kiuchi K (1988). Homospecific activity (activity per enzyme protein) of tyrosine hydroxylase increases in parkinsonian brain. J Neural Transm.

[CIT0024] Melamed E, Hefti F, Wurtman RJ (1982). Isr Compensatory mechanisms in the nigrostriatal dopaminergic system in Parkinson’s disease: studies in an animal model. J Med Sci.

[CIT0025] Chen CM, Yin MC, Hsu CC (2007). Antioxidative and anti-inflammatory effects of four cysteine-containing agents in striatum of MPTP-treated mice. Nutrition.

[CIT0026] Kieburtz K, Wunderle KB (2013). Parkinson’s disease: evidence for environmental risk factors. Mov Disord.

[CIT0027] Goldman SM (2014). Environmental toxins and Parkinson’s disease. Annu Rev Pharmacol Toxicol.

[CIT0028] de Haan JB, Bladier C, Griffiths P (1998). Mice with a homozygous null mutation for the most abundant glutathione peroxidase, Gpx1, show increased susceptibility to the oxidative stress-inducing agents paraquat and hydrogen peroxide. J Biol Chem.

[CIT0029] Bai X, Wey MC, Fernandez E (2015). Rapamycin improves motor function, reduces 4-hydroxynonenal adducted protein in brain, and attenuates synaptic injury in a mouse model of synucleinopathy. Pathobiol Aging Age Relat Dis.

