# Supplementary material for: Neurochemical and motor changes in mice with combined mutations linked to Parkinson’s disease
Source: Pathobiol Aging Age Relat Dis. 2017 Jan 5;7(1):1267855. doi: 10.1080/20010001.2017.1267855 (PMC5328310; doi:10.1080/20010001.2017.1267855)
Supplement: PBA_33852_Bai_Supplementary_figure.docx [file zpba_a_1267855_sm4878.docx]

**Supplemental Figure**


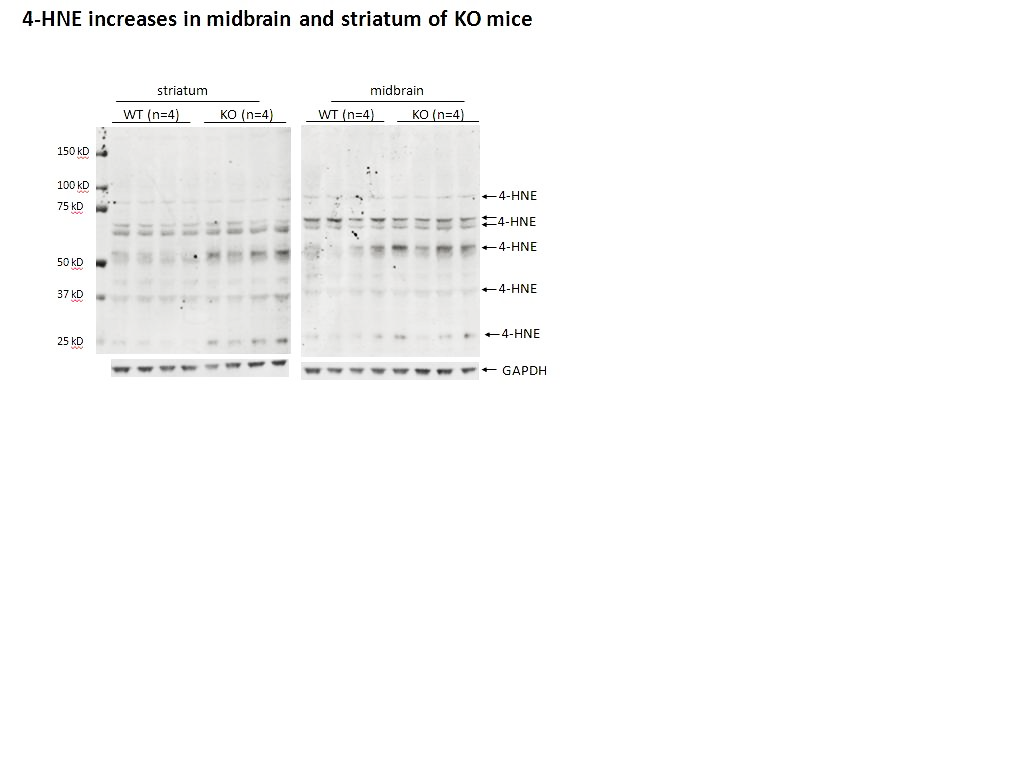


S1


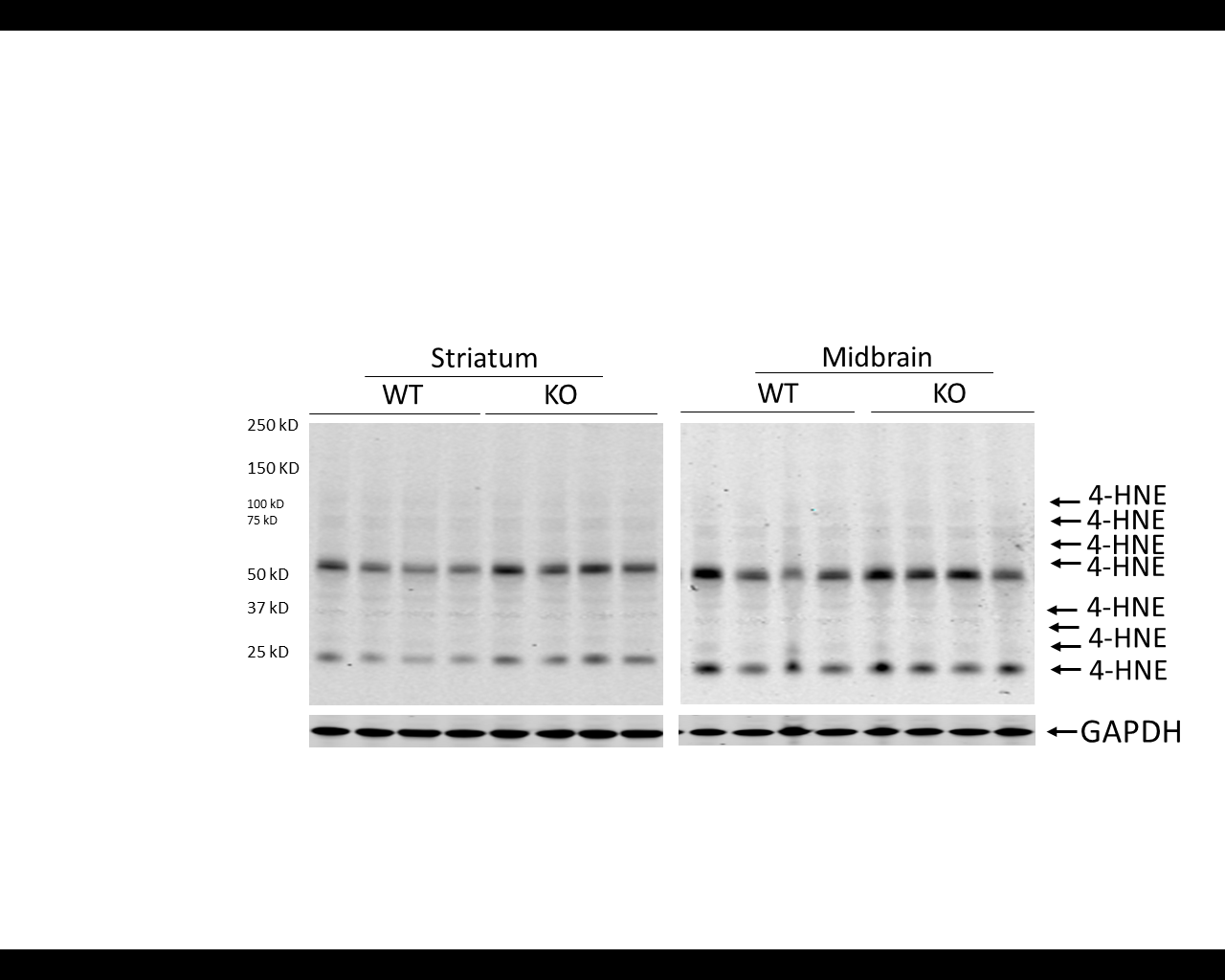


S2

**Supplemental Figure 1. Representative immunoblots of protein adducted to 4-HNE in striatum and midbrain.** S1. Blots prepared from mice sacrificed at 6 months of age. S2. Blots prepared from mice sacrificed at 18 months of age.
